# Acute Rickettsiosis Triggering *Plasmodium vivax* Relapse in a Returned Traveler: A Case Report and Clinical Review of Travel-Related Coinfections

**DOI:** 10.3390/pathogens14080768

**Published:** 2025-08-03

**Authors:** Ruchika Bagga, Charlotte Fuller, Kalsoom Shahzad, Ezra Bado, Judith Joshi, Dileesha Fernando, Amanda Hempel, Andrea K. Boggild

**Affiliations:** 1 Department of Laboratory Medicine & Pathobiology, University of Toronto, Toronto, ON M5S 1A1, Canada; 2 Department of Pathobiology and Laboratory Medicine, Schulich School of Medicine and Dentistry, Western University, London, ON N6A 5C1, Canada; 3 Victoria Hospital, London Health Sciences Centre, London, ON N6A 5W9, Canada; 4 DeGroote School of Medicine, McMaster University, Hamilton, ON L8S 4L8, Canada; 5 Temerty Faculty of Medicine, University of Toronto, Toronto, ON M5S 3K3, Canada; 6 Tropical Disease Unit, Toronto General Hospital, 200 Elizabeth Street, 13EN-218, Toronto, ON M5G 2C4, Canada; 7 Department of Medicine, University of Toronto, Toronto, ON M5S 3H2, Canada; 8 Institute of Medical Science, University of Toronto, Toronto, ON M5S 1A8, Canada

**Keywords:** eschar, fever in the returned traveler, malaria, relapsing malaria, rickettsioses

## Abstract

Given the overlap of epidemiological and clinical presentations of both rickettsioses and malaria infections, diagnostic testing where malaria is confirmed or excluded, without subsequent rickettsial testing, specifically in the case of *Plasmodium vivax* or *P. ovale* infection, may mask the possibility of relapse. A lack of clinical suspicion of co-infections, absence of knowledge on the geographic distribution of diseases, and lack of availability of point-of-care diagnostic testing for other tropical diseases can often lead to missed diagnosis or misdiagnosis of common tropical infections, including rickettsioses. We herein describe a case of confirmed intercurrent rickettsial and *P. vivax* infection, with the former potentially triggering a relapse of the latter in a febrile traveler returning to Canada from South America, and review the literature on tropical coinfections in returning travelers.

## 1. Introduction

Fever in returned travelers is a relatively common clinical presentation, affecting approximately 3–5% [1] of international travelers. The differential diagnosis is shaped by several factors, including the duration and destination of travel, the nature of activities undertaken, and the time elapsed since return [2]. Certain infectious etiologies may be associated with pathognomonic findings such as characteristic rashes or eschars, underscoring the critical importance of a detailed travel history and comprehensive physical examination.

Malaria [3] remains the most frequently identified cause of fever in this population, and most countries have well-established protocols to facilitate prompt diagnosis through microscopy (thin and thick smears), rapid diagnostic tests (RDTs), and/or molecular analyses such as polymerase chain reaction (PCR) or loop-mediated isothermal amplification (LAMP) techniques. In contrast, other tropical infections often remain under-recognized [4,5]. This gap is driven by limited clinical suspicion, insufficient knowledge of disease epidemiology, and a lack of access to point-of-care testing (POCT) for non-malarial pathogens.

Co-infections in particular may be missed, and should be actively considered in regions where vector distributions overlap. Documented examples include dual infections with arboviruses such as dengue and chikungunya [6], dengue with malaria [7], and even concurrent enteric infections like *Salmonella* and *Shigella* [8]. Additionally, intercurrent infections have been implicated in the reactivation of latent infections, such as *Plasmodium vivax* relapse triggered by other systemic illnesses [9,10]. If testing stops at the malaria diagnosis, the triggering infection may be missed entirely. We herein report a case of *Rickettsia*-associated febrile illness and intercurrent *P. vivax* infection, the latter of which may have represented relapse triggered by acute rickettsiosis in a returned traveler from South America.

## 2. Clinical Case

**Figure 1:**
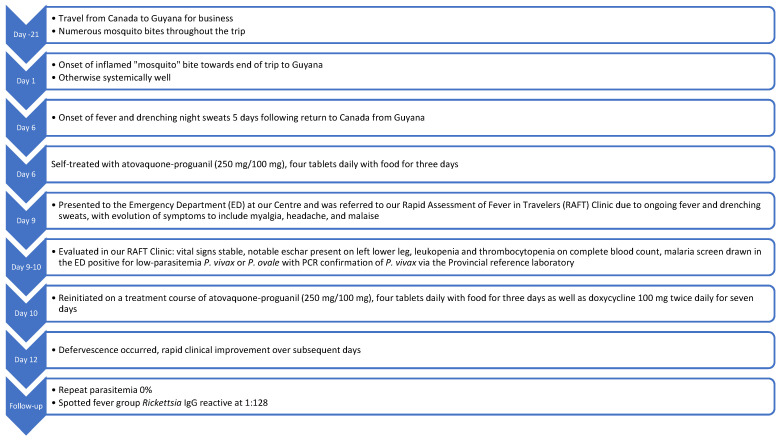
Timeline of symptom evolution and treatment.

A 55-year-old male presented to a Canadian Rapid Assessment of Febrile Travelers (RAFT) clinic with high-grade fever, malaise, and myalgias five days after returning from a three-week business trip to Guyana (Figure 1). The patient, a miner, reported extensive mosquito exposure while residing in a remote work camp. He had received standard immunizations and typhoid vaccination but did not take malaria chemoprophylaxis.

His past medical history was notable for previous episodes of *P. vivax* malaria, treated without radical cure (i.e., without primaquine). During this most recent trip, he recalled a particularly inflamed “mosquito bite” near the end of his stay in Guyana, which evolved into a necrotic lesion but was initially asymptomatic. Five days post-return, he developed fevers with drenching sweats and empirically self-initiated treatment doses of his atovaquone-proguanil prescribed pre-departure in Canada for antimalarial prophylaxis (250 mg/100 mg, four tablets daily with food for three days) beginning 3 days prior to consultation in our unit. At symptom onset, he denied associated myalgias, arthralgias, or rash, but noted retro-orbital pain. The necrotic bite remained inflamed and tender throughout this systemic febrile illness. Symptoms did not abate with his empiric course of antimalarial treatment, and continued to progress with ongoing fever, malaise, and development of myalgia.

**Figure 2:**
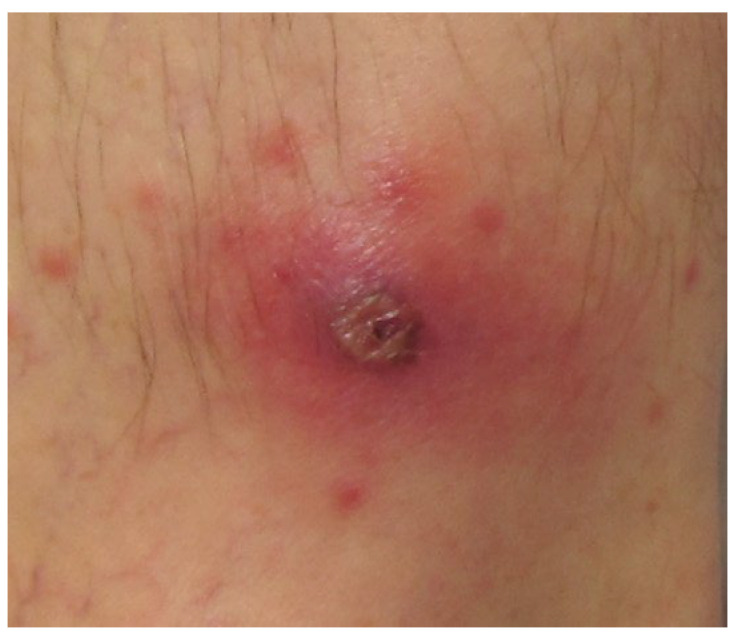
Necrotic eschar representative of the lesion reported by the patient described herein.

On presentation in our unit, vital signs were stable and he was afebrile. There was no evidence of rash, jaundice, hepatosplenomegaly, or lymphadenopathy. The patient was neurologically intact without evidence of nuchal rigidity or jolt accentuation of headache. A black eschar was noted on the left leg (Figure 2). The remainder of the physical examination was unremarkable.

Initial investigations revealed anemia (Hb 136 g/L; normal range 140–160 g/L), thrombocytopenia (platelets 104 × 10^9^/L; normal range 150–400 × 10^9^/L), and leukopenia (white blood cell [WBC] count 3.5 × 10^9^/L; normal range 4–11 × 10^9^/L; Beckman Coulter, Mississauga, ON, Canada). Electrolytes and liver function tests including bilirubin and hepatic transaminases were within normal limits, as was renal function. Malaria screening (BinaxNOW rapid diagnostic test [RDT; Alere, Montreal, QC, Canada] and smear) revealed low-level parasitemia consistent with *P. vivax* or *P. ovale* (<0.1%). Pan-*Plasmodium* real time PCR followed by species-specific duplex real time PCR [11] performed at our provincial reference laboratory confirmed *P. vivax*. The patient was continued on an additional 3-day course of atovaquone-proguanil with food and also started on oral doxycycline 100 mg twice daily for 1 week [12] for presumed rickettsial infection while awaiting serology. He declined the primaquine radical cure due to reported intolerance in the past and recognized the importance of ongoing vigilance for febrile illness in the future, given the potential for recurrent relapse. Monospot (heterophile antibody) for the detection of acute mononucleosis and blood cultures for the detection of bacteremia, notably Enterobacteriaceae including *Salmonella* spp., were negative. Within 48 h of beginning a second course of atovaquone-proguanil and first course of doxycycline, he defervesced. Spotted fever group (SFG) rickettsial IgG drawn upon presentation to our unit and performed at our Provincial reference laboratory later returned reactive at a titre of 1:128 (reactive ≥ 1:64) [13]. This assay has a turnaround time of 10 business days from receipt at the reference laboratory and is based on the Focus Diagnostics *Rickettsia* Indirect Immunofluorescence Antibody (IFA) IgG kit, which is a duplex assay that “provides semi-quantitative detection of IgG antibodies to either SFG rickettsiae (based on a *R. rickettsii* antigen)” or typhus group (TG) “rickettsiae (based on a *R. typhi* antigen)” [13].

Subsequent malaria testing at the patient’s next follow-up visit revealed a parasitemia of 0%. Fully convalescent serologic testing for rickettsioses was not sent as the patient had departed for further travel and recognized the lack of clinical value of such testing. Given the clinical findings of a painful eschar, very low *P. vivax* parasitemia, prior *P. vivax* infection without radical cure, and rapid response to doxycycline, this likely represented a primary *Rickettsia* spp. infection that possibly triggered a relapse of *P. vivax*.

## 3. Discussion

### 3.1. Rickettsia in South America

Rickettsial diseases are globally distributed [14] yet frequently underdiagnosed [15,16] due to nonspecific clinical manifestations and limited access to timely diagnostics. In regions such as South America, where multiple spotted fever rickettsiae circulate, early clinical recognition is further complicated by overlapping symptomatology with other tropical febrile illnesses. Based on our patient’s epidemiology and given the mild nature of his clinical presentation, we presume this to be a case of *R. parkeri* infection, rather than *R. rickettsia* infection.

Several Spotted fever group rickettsiae have emerged across South America as important zoonotic agents associated with eschar formation. *R. parkeri* strain Atlantic rainforest has been detected in *Amblyomma ovale* ticks and domestic dogs in Brazil, highlighting its potential for human infection and eschar-associated febrile illness [16]. In their seroprevalence study of anti-*Rickettsia* spp. antibodies in humans and dogs in the Paraná state of southern Brazil, Kmetiuk and colleagues noted seropositivity in 12% of people and 22% of domestic dogs, while also identifying *Rickettsia sanguineus* and *R. parkeri* in ticks collected from the dogs [17]. Likewise, *R. amblyommii*-like organisms have been reported in *Amblyomma cajennense* complex ticks in French Guyana, with documented links to localized necrotic eschars in humans [18]. A recent molecular survey in Colombia also confirmed the presence of *Rickettsia* spp. in ticks infesting wild animals across multiple ecosystems, underscoring the wide environmental range of eschar-producing rickettsioses [16]. In their systematic review and modeling analysis, Zhang and colleagues note that the major spotted fever group (SFG) rickettsioses throughout South America include the aforementioned *R. rickettsii*, *R. parkeri*, and *R. amblyommii*; however, *R. felis*, *R. conorii*, *R. massiliae*, and *R. aeschlimannii* are present in certain ecologies and a range of vectors as well [19]. In terms of conclusive clinical disease in humans, the most causally related species in South America are clearly *R. rickettsii*, *R. parkeri*, and *R. felis* [19].

These pathogens are frequently underrecognized due to lack of clinician awareness, underutilization of molecular diagnostics, and geographic underreporting [14]. Therefore, febrile illness with eschar in returned travelers from South America should prompt consideration of Spotted fever group rickettsioses, especially when supported by epidemiologic context.

Spotted fever group rickettsioses are typically diagnosed clinically through recognition at the bedside of compatible epidemiology, risk factors, and pathognomonic features such as eschar. Confirmatory microbiological diagnosis usually rests on serological confirmation [5], which may be delayed and may be initially negative in the first week of disease. A four-fold rise in serologic titre between acute presentation and convalescence or a very high single serologic titre would be considered confirmatory, with sensitivity of the former approaching 100% [13]. Moreover, positive molecular diagnostic testing—notably, PCR of eschar fluid, blood, or tissue biopsy—is also considered confirmatory from a microbiological standpoint. At our National Reference Laboratory, an in-house “real-time PCR specific for the 17 kDa antigen gene and citrate synthase gene of the *Rickettsia* genus” has been developed for use [13]; however, the prolonged turnaround time of 21 days from receipt at the Provincial reference laboratory limits the clinical utility of such a test. As illustrated in this case, the absence of a rapid bedside microbiological diagnostic necessitates reliance on clinical diagnosis and empiric treatment of rickettsiosis in the setting of compelling presentation, physical examination findings, and epidemiology [20].

### 3.2. Rickettsia and Malaria Co-Infection

In areas of overlapping transmission [21], co-infections may alter the disease course, clinical recognition, and treatment outcomes. *Rickettsia* and malaria occur in overlapping regions but there is limited literature on coinfections and reciprocal impact on disease severity [16]. Our case emphasizes the importance of a syndromic and epidemiologically informed diagnostic approach in febrile returned travelers, and consideration of multiple diagnoses. Without clinical suspicion, patients may be misdiagnosed with malaria alone without recognition or treatment of co-infection.

Literature on *Rickettsia*-malaria coinfection is sparse. Tay and colleagues [22] documented the first molecular evidence of *Rickettsia* spp. RF2125 co-infection in a *P. vivax*-positive patient in Malaysia. Similarly to our case, that patient had low parasitemia and febrile symptoms mimicking malaria, highlighting the potential for missed rickettsioses in malaria endemic areas. Their findings emphasized the need for broader diagnostic workup and greater clinical awareness of coinfections, particularly where there is overlapping vector exposure and shared ecological niches.

Mediannikov and colleagues [23] demonstrated seasonal and geographic overlap of *Rickettsia felis* and *Plasmodium* infections in sub-Saharan Africa. In their study of over 3000 patients, coinfection rates reached as high as 23% in Senegal. Their findings suggest that empirical fever-management strategies in malaria-endemic regions should be revisited, as doxycycline-based malaria prophylaxis may also mitigate undiagnosed rickettsioses [12]. These data underscore the overlapping transmission patterns of malaria and rickettsial pathogens and their potential clinical synergy or interaction, particularly in returned travelers.

### 3.3. Rickettsia as a Trigger for Malaria Relapse

Beyond coexistence, rickettsial infections may also influence the clinical expression of malaria. Both *P. vivax* and *P. ovale* form latent hepatic forms known as hypnozoites that can activate at later stages to cause a relapse of infection [24]. While the stimulus for activation is not entirely understood, it has long been hypothesized that the febrile illness cause by new malaria infection is a trigger for activation, as it signals the presence of the vector [25]. However, febrile illness from other infections may also be a trigger for reactivation, with historical evidence for typhoid, relapsing fever, trench fever and endemic typhus having been described [26]. It has been proposed that the host responses triggered by systemic parasitic or bacterial infections, such as *Rickettsia*, may therefore be potential triggers although this is difficult to study in diseases not associated with large epidemics [26]. The case described here is consistent with a relapse of *P. vivax* possibly triggered by the febrile illness associated with acute *Rickettsia* infection, although the data available to us are insufficient to conclude a direct causal link.

Doxycycline’s activity against both rickettsial and plasmodial pathogens supports its use in empiric regimens when co-infection is suspected. However, definitive cure of *P. vivax* and *P. ovale* still necessitates effective blood schizonticide (e.g., chloroquine, atovaquone-proguanil, artemether-lumefantrine) administration followed by hypnozoite eradication with a tissue schizonticide such as primaquine or tafenoquine, underscoring the importance of patient counseling and G6PD testing where applicable [24]. Our patient was uninterested in radical cure given prior intolerance of primaquine, and as such was counseled on the importance of future vigilance for febrile illness that would warrant urgent exclusion of malaria. In such a patient, for whom the risks and benefits of radical cure are understood, and for whom medical care is rapidly accessible, vigilant watchful waiting and presentation for care upon new onset of fever is a reasonable, mutually agreeable strategy.

Clinicians should maintain high vigilance for atypical malaria presentations, particularly in patients with prior *P. vivax* or *P. ovale* infections and recent vector exposures. Cases with very low parasitemia and syndromes not fully explained by malaria may represent reactivation in the context of another illness. Eschars, spotted fevers, cytopenias, or lack of full response to antimalarial monotherapy may be clinical clues suggesting *Rickettsia* as an intercurrent infection. Furthermore, clinicians treating rickettsial infections in patients with prior *P. vivax* or *P. ovale* infections should monitor for the development of relapsed malaria during or after acute illness.

## 4. Conclusions

In conclusion, we present a case consistent with a rickettsial infection that possibly triggered a *P. vivax* relapse based on compatible temporal, clinical, and laboratory evidence, including very low *P. vivax* parasitemia (<0.1%) and the lack of radical cure with a tissue schizonticide such as primaquine during prior malaria episodes. However, primarily acquired *P. vivax* infection or relapse precipitated by some other stressor (including travel itself) cannot be definitively excluded based on the available data. The review of malaria and *Rickettsia* co-infection highlights the importance of excluding co-infections in travelers diagnosed with malaria, particularly when symptoms suggest an alternative etiology. Clinicians should also consider malaria relapse triggered by intercurrent infections in appropriate settings. Appropriate and timely treatment may be lifesaving in such scenarios, and empiric therapy may be necessary given the lengthy turn-around time for diagnostic testing.

## Data Availability

The original contributions presented in this study are included in the article. All data are found herein. Further inquiries can be directed to the corresponding author.
